# Medicine Men of the World

**Published:** 1889-04-20

**Authors:** 


### Medicine Men of the World.

In a previous article reference has been made to the curious fact that the great African tribe, the Bakmoins, had in some mysterious way got hold of the European idea of inoculation for smallpox. An equally interesting fact may be noted with respect to the Pawnee Indians of North America, who seem to have possessed a vague but unmistakable idea of the efficacy of one attack of smallpox to afford immunity from another. Whether this was a matter of their own observation, or whether they had borrowed the notion from the white men from whom they got the disease, we cannot say; but travellers affirm that no sooner does the dread malady make its appearance among the Pawnees than they all begin to dot their bodies all over with smallpox marks, seemingly in the conviction that if they can only impose on the malignant spirit who is directing the malady the belief that they have been attacked once, they are not likely to suffer from the outbreak. And seemingly this was a common practice among these poor savages long before civilised peoples had begun to think about artificial means of inducing mild forms of smallpox.

This fell disease, however, is one of the few against which, as a rule, the barbarians never pretend to have a specific. Catlin says that the destructive ravages of this most fatal disorder among the Indians has been beyond the knowledge and almost beyond the belief of the civilised world. "Terror and dismay are carried with it, and awful despair, in the midst of which they plunge into the river when in the highest state of fever and die in a moment, or dash themselves from precipices, or plunge their knives to their hearts to rid themselves from the pangs of slow and disgusting death." How is it that among the many phases of self-sacrifice, of which scientific men have shown themselves capable, nobody has ever heard of a vaccination mission to these and other unfortunate savage peoples, some of whom have been all but annihilated by the fell swoop of this dread outrider of civilisation? Perhaps it is that, as a rule, little has been heard of these fearful outbreaks till they have exhausted themselves.

Of course, such specifics for disease or precautions against it as have just been alluded to are common among barbarians. There superstitious trickery is just what would be expected; but many people who do not stand very high in the esteem of the civilised world appear to have anything but contemptible notions of medicines. The medicine man of the Turkistan parts, for instance, is quite a respectable practitioner. His first care, says Schuyler, is to study your general appearance and ask you about your temperament. You must, in his belief, belong to one of four classes, and after he has elaborated in his own mental depths some theory as to the requirements of a patient of your special symptoms and peculiar temperament, he will pull a bag out of his pocket, or untie the scarf which serves him for a girdle, and open an assortment of drugs in twisted bits of paper, perhaps tasting or smelling to find the right one, and having chosen the proper medicine, will give you the usual directions about doses and diet. "The medicaments employed by Central Asiatic," continues Dr. Schuyler, "are, in general, very simple, being, for the most part, vegetable substances, but few mineral matters and minerals being used. They are usually taken simply in the form of powders or decoctions, and when a mixed medicine is used the physician delivers the substances to the patient, and allows him to mix them himself." This is done partly to save the doctor trouble, and partly as a precaution against the suspicion of poisoning in the event of results not being satisfactory.

This writer quotes from a well-known authority a statement to the effect that medicines are pretty much the same in all Mussulman countries, and are, in fact, all of Arabic origin, or nearly so. If there are any exceptions, it is in the case of some vegetable substances not easily procurable in some of the regions to which Mohammedanism has spread. It affords an amusing idea of Oriental medicine to be told that in such cases other vegetable substances have been substituted as nearly the same in appearance as they could possibly be procured, even though their active properties may be entirely different. The European vegetable drugs that have from time to time been imported to the East are all of them said to have been familiar there from time immemorial.

It need hardly be said that Mussulmans, as a rule, will not submit to medical treatment at the hands of unbelievers; but European doctors nevertheless often find their skill very useful in travelling in Mohammedan countries. The devotees of the Prophet will not take their medicines, but they are often enough glad to consult a dog of an unbeliever in many matters of health and well-being, in which they appear to have exhausted the resources of native medicine men. Sir Alexander Burnes, in the history of his diplomatic relations with Cabul, relates an amusing instance of the kind. The brother of Moorad Bey, the chief of the Koondooz, had become dim-eyed, and in spite of all that the native doctors could do for his relief appeared to be in danger of losing his eyesight altogether. Application was made, therefore, on his behalf to the English, and Sir Alexander deputed Dr. Ward and Lieutenant Wood to see what could be done for the poor old grandee. Everything was tried that seemed likely to be of service to him, but, alas! there was no cure for old age, and the European doctors were bound to admit that the case was beyond their skill. A final interview at length took place between the great men and the English medicine men, of whom he had hoped so much. "The scene," says Sir Alexander Burnes, "was a strong mixture of the pathetic and the ludicrous. I could not help sympathising sincerely with the poor old man and his son, a fine lad of fifteen, who shared deeply in his father's grief; but every broad-faced Uzbek about the room, seeing his chief in tears, thought it incumbent on him to blubber a little also, and the wry faces some of them made in an attempt to look melancholy were perfectly irresistible. I was obliged to bury my face in my sleeve, and hope I, too, got credit for crying a little."

